# Effects of Cocoa Polyphenols and Dark Chocolate on Obese Adults: A Scoping Review

**DOI:** 10.3390/nu12123695

**Published:** 2020-11-30

**Authors:** Hasmiza Halib, Amin Ismail, Barakatun-Nisak Mohd Yusof, Naomi Osakabe, Zulfitri Azuan Mat Daud

**Affiliations:** 1 Department of Dietetics, Faculty of Medicine and Health Sciences, Universiti Putra Malaysia, Serdang 43400, Malaysia; hasmizahalib@unisza.edu.my (H.H.); bnisak@upm.edu.my (B.-N.M.Y.); 2 School of Nutrition and Dietetics, Faculty of Health Sciences, Universiti Sultan Zainal Abidin, Kuala Terengganu 21300, Malaysia; 3 Department of Nutrition, Faculty of Medicine and Health Sciences, Universiti Putra Malaysia, Serdang 43400, Malaysia; aminis@upm.edu.my; 4 Research Center of Excellent (RCoE) Nutrition and Non-communicable Diseases, Faculty of Medicine and Health Sciences, Universiti Putra Malaysia, Serdang 43400, Malaysia; 5 Department of Bio-Science and Engineering, Shibaura Institute of Technology, Saitama 337-5780, Japan; nao-osa@shibaura-it.ac.jp

**Keywords:** polyphenols, flavanols, cocoa, dark chocolate, obesity, obese adults

## Abstract

Obesity remains a major public health problem due to its increasing prevalence. Natural products have become common as adjunct therapeutic agents for treating obesity and preventing metabolic diseases. Cocoa and its products are commonly consumed worldwide. Dark chocolate, a rich source of polyphenols, has received attention lately for its beneficial role in the management of obesity; however, conflicting results are still being reported. This scoping review aims to provide a comprehensive understanding of the existing literature on the relationship and effects of cocoa and dark chocolate intake among obese adults. We searched multiple databases for research investigating the consumption of cocoa and/or dark chocolate in managing obesity among adults. This review includes epidemiological and human studies that were published in English over the last 10 years. Our review of the current literature indicates that epidemiological and human trials with obese adults have shown inconsistent results, which may be due to the different populations of subjects, and different types of cocoa products and doses used for intervention. Studies among obese adults are mainly focusing on obese individuals with comorbidities, as such more studies are needed to elucidate the role of cocoa polyphenols in weight control and preventing the risk of chronic diseases among obese individuals without comorbidities as well as healthy individuals. Careful adjustment of confounding factors would be required. The effects of cocoa and dark chocolate intake on obese adults were discussed, and further research is warranted to identify the gaps.

## 1. Introduction

Obesity has become a public health problem globally due to its alarmingly high rate and increasing prevalence, as well as the role it plays in the occurrence of many chronic diseases [1]. Overweight and obesity are defined as abnormal or excessive fat accumulation that may impair health [2]. The rate of obesity has increased globally, tripling since 1975. More than 1.9 billion adults aged 18 years and older were reported to be overweight in 2016; of these, 600 million adults were obese. In addition, the prevalence of overweight and obesity was also found to be high in low- and middle-income countries, especially in urban areas [3].

A lack of physical activity and poor nutrition have been documented to contribute significantly to the increased prevalence of obesity and its associated complications [4]. Obesity contributes to many impacts, including physical, social, psychological, and, most importantly, medical [5]. Substantial evidence shows that overweight and obese people are more likely to develop a variety of chronic diseases, including hypertension, type 2 diabetes, and cardiovascular disease (CVD) [6]. Obesity is often characterized as low-grade chronic inflammation with irregular inflammatory response, weak antioxidant capability, and low insulin sensitivity, resulting in inflammation, oxidative stress, and insulin resistance [7]. Adipose tissue inflammation plays a crucial role in causing obesity-related metabolic complications, including the development of insulin resistance [8]. In response to the increased obesity prevalence, numerous efforts have been made and strategies implemented to tackle this problem. Lifestyle interventions such as a calorie-deficit diet and exercise are often difficult or unsuccessful [9]. Thus, the use of natural products as therapeutic agents for the treatment of metabolic disorders has become a common alternative or complementary method for the treatment of obesity.

Polyphenols are present in several widely consumed foods, such as fruits and vegetables, and in beverages, such as coffee, tea, and wine, as well as dry legumes, chocolate and cocoa products. Over the past two decades, polyphenols have gained considerable attention mainly due to their antioxidant and their potential role in the prevention of many diseases, such as CVD, cancer, diabetes, and other oxidative stress-related diseases [10,11]. Polyphenols, classified according to their chemical structure, include phenolic acids, flavonoids, stilbenes, lignans, and tannins [12]. Flavonoids are the most abundant type of polyphenol in food. Flavonoids can be classified into groups: flavonols, anthocyanidins, flavones, flavanones, flavanonols, and isoflavonoids. Cocoa and its products, a commonly consumed food around the world, are a source of polyphenols [13] that show higher phenolic content and total antioxidant capacity than tea and red wine [14]. Cocoa polyphenols consist primarily of flavanols (epicatechin, catechin, and procyanidins) and a flavonol (quercetin). Other polyphenols have also been reported to be present in small quantities, such as anthocyanins, phenolic acids, and stilbenes [15]. The benefits of cocoa and cocoa products depend on polyphenol content. Cocoa flavanols can be found in dark chocolate, with a content estimated to be five times higher than that in milk chocolate [16]; the content of catechin and epicatechin is approximately 20 times higher than in tea.

Several in vitro and in vivo studies have shown that polyphenols, with antioxidant, anti-inflammatory, and anti-obesity properties, can boost energy expenditure and thermogenesis, and lessen oxidative stress and inflammation, while supporting weight loss management [17]. The popularity and regular consumption of chocolate and cocoa products and their postulated role in improving obesity have made them the subject of several research studies. Studies that elucidate these effects are needed to explore the possible therapeutic mechanism of cocoa in obesity.

The objective of this scoping review was to summarize the available evidence regarding the effects of cocoa and dark chocolate consumption on glucose profiles, lipid profiles, oxidative markers, inflammatory markers, and body weight regulation among obese adults. What do we know about such effects and to what extent has the available research explored these effects on an obese individual?

## 2. Method

The review was conducted based on a framework by Arksey and O’Malley [18]. The guidelines used to record the review process were based on Preferred Reporting Items for Systematic Reviews and Meta-Analysis (PRISMA). This scoping review includes a broad collection of articles on experimental and observational studies and controlled trials. The framework comprises five stages: identifying the research question, identifying relevant literature, selecting literature, charting data and collating, summarizing, and reporting results. The framework charts, collects, and summarizes the known literature on a particular topic, and attempts to classify all of the literature irrespective of its content.

### 2.1. Identifying Research Question

The research question for this scoping review was, what do we know about the effects of cocoa polyphenols/dark chocolate on the metabolic profiles of obese adults, and to what extent has the clinical effectiveness been explored?

### 2.2. Identifying Relevant Literature

Articles from 2008 through 2019 on studies related to cocoa polyphenols and their effects on obesity were searched. Articles were retrieved based on keywords using multiple electronic databases (PubMed, Scopus, and ScienceDirect). The medical subject heading (MeSH) terms “cocoa” OR “cocoa polyphenol” OR “cocoa flavanol” OR “dark chocolate” AND “obese” OR “obesity” OR “body mass index” OR “Body Mass Index (BMI)” OR “body fat” OR “body fat” were utilized, and articles published in English from 2008 to 2019 were included. Study designs included in this scoping review include randomized controlled trials and observational studies that specifically looked into the effects of cocoa/dark chocolate on obese/overweight adults. Studies that did not meet the selection criteria or were not related to the research questions and duplicate publications were excluded. Further, several references that were obtained in full text were examined for additional material.

### 2.3. Selecting Literature

**Figure 1:**
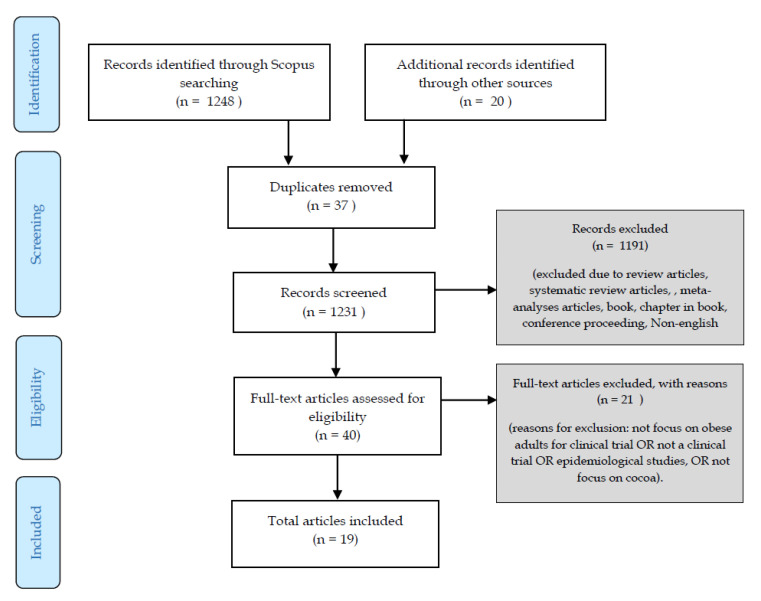
Summary of the selection of publications.

The full references of articles retrieved from the databases were downloaded and input manually into Excel. Two reviewers screened the articles for inclusion, and any discrepancies were resolved by the third reviewer. A total of 1248 articles were identified; 37 duplicates were removed, 1211 titles and abstracts of articles were screened based on the inclusion and exclusion criteria (Table 1), and a final total of 19 articles were read in full text. The article selection process is shown in Figure 1.

### 2.4. Charting Data

A narrative approach and an analytical approach were used to extract data from papers that better addressed the research aims based on Arksey and O’Malley (2005) [18]. Each article was summarized in a scoping table and categorized by two authors into authors, country, year of publication, aim, study design, participants, and outcomes. An overview of the included material is provided in Table 2. Comments, letters, conference abstracts, reviews, short communications, meta-analyses, and animal studies were excluded.

### 2.5. Collating, Summarizing, and Reporting Results

Data extraction focused on variables or findings related to blood glucose profiles, lipid profiles, oxidative and inflammatory markers, body weight, body fat, and other measures related to obesity outcome. Each article was read thoroughly and all information regarding study design, study duration, study site, type and dosage of treatment, study population, and measurement of interest and major findings was recorded. The full-length articles were reviewed by at least two of the authors (H.H. and Z.A.M.D. or A.I.). Findings relevant to obesity or treatment (fasting blood glucose, blood lipids, blood pressure, body mass index, body fat, and waist circumference) were recorded.

## 3. Results

### 3.1. Articles Identified

A total of 19 publications that met all the inclusion and exclusion criteria were included for final review: 14 clinical studies (Table 2) and 5 observational studies (Table 3). All included studies were conducted in western countries. A majority of the studies were conducted in the US (7) and Spain (4); two were conducted in the UK and one each in Australia and Mexico.

### 3.2. Study Populations

Of the 14 clinical studies, 6 were focused on obese subjects with risk factors for cardiovascular disease [19,20], 2 on obese subjects with co-existing type 2 diabetes mellitus [21,22], 1 on postmenopausal women [23], 2 on obese individuals without chronic disease [24,25], and 3 on a combination of cocoa supplementation with exercise [26] or an energy-restricted diet [27,28].

### 3.3. Epidemiological Studies

Cross-sectional epidemiological studies conducted in the US among adults participating in the Nutrition Health and Nutrition Examination Survey (NHANES) between 1999 and 2004 reported that chocolate consumers had significantly lower weight, triglyceride (TG) levels, and C-reactive protein (CRP) levels, and significantly higher high-density lipoprotein (HDL-c) levels than nonconsumers. Higher chocolate intake among Europeans has also been shown to be correlated with lower levels of total and central fat, as measured by BMI, body fat derived from skin folds, bioelectrical impedance analysis (BIA), and waist circumference regardless of potential confounders such as sex, age, sexual maturation, total energy, saturated fat, fruit and vegetable intake, and physical activity. The results remained unchanged even after adjusting for tea and coffee intake and removing overweight and obese adolescents [29].

Longitudinal research suggests that ingesting flavonoids can have a beneficial long-term effect on weight maintenance. A study found that dietary consumption of catechins present in fruits, vegetables, chocolate, and tea was inversely correlated with BMI in women followed over 14 years [30]. A recent large cross-sectional study using data from NHANES that evaluated the association between flavonoid consumption and risk factors for obesity and obesity-related inflammation reported an inverse association between flavonoid consumption and obesity in men and women as assessed by BMI and waist circumference (WC) even after adjusting for potential confounders (e.g., sex, age, race, physical activity, energy intake, and smoking status). The study found that adults in the highest quartile of flavonoid intake had significantly lower BMI and smaller WC. The study also showed an inverse association between flavonoid consumption and C-reactive protein level, a marker for inflammation [31].

Golomb et al. [32] reported an inverse association between the frequency of chocolate consumption and BMI in 1000 adults aged 20 to 85 years. The study reported that the frequency of chocolate consumption was linked to higher caloric and saturated fat intake, but positively related to BMI. The association remained even after adjusting for age, gender, physical activity, and dietary intake and was not explained by caloric intake from the chocolate. However, the results were cross-sectional, so the causality of the observed correlation cannot be presumed. In addition, the use of a food frequency questionnaire alone could lead to bias, and the study also did not distinguish between types of chocolate being consumed. More comprehensive prospective epidemiological studies are therefore required to validate these cross-sectional findings.

In contrast, a prospective and cross-sectional study done on the association between chocolate intake and body weight involving 16,000 persons randomly selected from four US communities found that frequent chocolate consumption was associated with significantly greater prospective weight gain over time. Increased BMI was found to be higher in participants who consumed chocolate more frequently, in a dose-response manner. However, this relation was attenuated after omitting participants with pre-existing obesity, in which case the correlation between chocolate consumption and lower weight did not extend to those without pre-existing illness. A cross-sectional analysis revealed the opposite result: chocolate consumption was reported to be associated with significantly lower BMI [33]. The discrepancy between prospective and cross-sectional results was likely because subjects diagnosed with obesity-related diseases tend to reduce their energy intake, especially energy-rich foods such as chocolate, to improve their prognosis, thus causing a reverse correlation between chocolate intake and BMI.

In conclusion, epidemiological studies present contradictory findings that could be due to insufficient adjustment of confounding factors and the type of chocolate being consumed, with different types, such as white, milk, and dark chocolate, not distinguished from each other. The polyphenol content in chocolate is not reported in all epidemiological studies because the polyphenol content in chocolate plays an important role in providing beneficial health effects; therefore, the studies were not able to assess the beneficial effects of different types of chocolate. Logically, the high caloric density of chocolate is supposed to increase the risk of weight gain, but different types of chocolate have different caloric content, thus contradictory findings are reported. Furthermore, these epidemiological studies could not draw causal inferences regarding the effects of chocolate consumption on body weight or body mass index. Also, these studies relied on self-reported data, mainly from dietary intake questionnaires, which might cause over- and underestimates in reporting dietary intake. Thus, more rigorous prospective epidemiologic studies need to be conducted in the future to provide more reliable data, especially regarding the consumption of chocolate or cocoa, with more information on the types of chocolate and the polyphenol content.

### 3.4. Intervention Studies

#### 3.4.1. Intervention Design and Dosage

The duration of intervention studies included in this review ranges from 1 day to 6 months (24 weeks). One study was conducted in an acute setting for 6 h and one study was conducted for 5 days. In terms of study design, 3 studies had a parallel design and 11 studies had a cross-over design. The sample size ranged from 14 to 84 participants. Two studies were specifically performed with women and twelve with both genders. Eight studies used cocoa beverage in powdered form, three studies used cocoa extract in capsule form, and five studies used dark chocolate. Three studies assessed the effects of different doses of polyphenols, ranging from 30 to 1000 mg. Table 2 provides an overall summary of intervention studies.

#### 3.4.2. Changes in Body Weight, BMI, Waist Circumference, and Blood Pressure

A total of 12 of 14 studies included in this review reported body weight and BMI as either a primary or secondary outcome measure. Two studies did not report any anthropometric measurements as the measured outcome [21,22]. Five studies did not find any changes in body weight and BMI. Two studies noted an increase in body weight. Three studies reported a reduction in body weight and BMI. Out of 14 studies, only six reported waist circumference as one of the outcomes. Only two studies noted smaller waist circumference in the treatment group. Blood pressure was measured in all studies; however, only six studies reported improvements in blood pressure [24,25,26,27,28,34].

A study done by Nickols-Richardson et al. [28] comparing the effects of incorporating dark chocolate plus a daily sugar-free cocoa beverage into an energy-restricted diet (ERD) and a non-chocolate snack plus a sugar-free cocoa beverage in women revealed reductions in body weight, BMI, waist and hip circumference, and body fat percentage after 18 weeks of the intervention in both groups at any interval and over time. Moreover, reductions were also noted for systolic and diastolic blood pressure and glucose and insulin concentration in both groups; however, no significant difference was found between the two groups. The limitation of the study was that it did not include a non-snack and non-beverage control; thus, further evaluation is needed.

Similar results were reported in a study done on the effects of daily consumption of ready-to-eat meals supplemented with cocoa extract in a hypocaloric diet in obese subjects, where the control group received only hypocaloric control meals. After 4 weeks of intervention, both groups had significantly improved body weight, BMI, waist circumference, and body fat. The concentrations of total cholesterol (TC), low-density lipoprotein cholesterol (LDL-c), high-density lipoprotein cholesterol (HDL-c), triglyceride (TG), and insulin were also found to be improved in both groups. Among studies that only used cocoa or dark chocolate without combining them with exercise and ERD, only one study noted a reduction in body weight and BMI [35]. The study reported that consumption of cocoa bean extract powder containing 80 mg of flavonoids for 4 weeks by obese subjects with borderline metabolic syndrome showed a reduction in body weight, waist circumference, glycemia, and TG. Blood levels of HDL-c also increased in the cocoa group. The study also examined oxidative damage biomarkers and noted that levels of malondialdehyde (MDA) and free carbonyls were significantly lower in the cocoa group compared to placebo. In addition, a study by Almoosawi et al. [24] of healthy obese subjects noted a significant difference in body weight between the placebo group and the group that consumed polyphenol-rich dark chocolate at the end of 4 weeks. The study also reported that fasting glucose and diastolic blood pressure (DBP) were reduced in the dark chocolate group, especially in subjects with higher BMI.

#### 3.4.3. Changes in Glucose and Lipid Profiles

All studies reported glucose and lipid profiles among their outcome measures. Eight studies reported no changes in glucose profiles (fasting blood glucose and insulin), six studies reported reductions in fasting blood glucose, Homeostatic Model Assessment for Insulin Resistance (HOMA-IR) values, and insulin levels. Only five studies noted reductions in TC, TG, and LDL cholesterol.

Six studies included in this review reported changes in glucose profiles (either fasting blood glucose (FBG), insulin level, HOMA-IR, or quantitative insulin sensitivity check index (QUICKI)), and these studies involved obese subjects with a risk of CVD or type 2 diabetes and healthy obese subjects. A study by Almoosawi et al. [25] investigating the effects of two doses of polyphenol-rich dark chocolate, 500 and 1000 mg, found that both doses were equally effective in reducing blood pressure and fasting blood glucose (FBG) in obese adults after 2 weeks of intervention. The study suggested that both doses have equal efficacy. The hormone cortisol was also measured, as it is linked to insulin resistance and increased fasting insulin [36], but no significant association was found between cortisol and cortisone levels and FBG and blood pressure (BP). Almoosawi et al. [24] conducted a further study on the effects of dark chocolate with 500 mg of polyphenols on biomarkers of glucose regulation in overweight and obese females for 4 weeks and reported that consumption of polyphenol-rich chocolate for 4 weeks reduced HOMA-IR and fasting glucose in females with BMI more than 25. In addition, in a longer intervention, Leyva Soto et al. [34] reported that HOMA-IR and FBG were significantly decreased after 6 months of daily intake of 2 g of flavonoid-rich chocolate, where the majority of subjects had normal blood pressure and high triglyceride and glucose levels. In contrast, a meta-analysis of cocoa and chocolate in terms of resistance or sensitivity to glucose and insulin is likely to show beneficial effects on insulin resistance (IR) but not on glucose levels [37]. One study included in this review also showed similar findings, where the intake of dark chocolate with a cocoa beverage improved IR but had no effect on fasting glucose [23].

#### 3.4.4. Changes in Oxidative and Inflammatory Markers

Out of the 14 clinical trials included in this review, only five studies measured either oxidative or inflammatory markers (CRP, high-sensitivity CRP (hsCRP), interleukin 6 (IL-6), intercellular adhesion molecule 1 (ICAM-1), oxidized low-density lipoprotein (oxLDL), and urinary-F2 isoprostane (MDA)) as part of their outcomes [20,22,27,38].

Two studies reported decreased inflammatory markers as measured by oxLDL. Khan et al. [19] conducted a randomized controlled trial with 42 volunteers and reported that oxLDL decreased significantly after consumption of 40 g of cocoa powder with 500 mL of skim milk compared to consumption of 500 mL of milk only [19]. Changes in inflammatory markers were also noted, with lower concentrations of ICAM-1 found after 4 weeks of consuming 40 g of cocoa with milk [20]. Another study done to determine the effects of the dose–response of cocoa flavanol intake on oxidative and inflammation markers found that as the cocoa flavanol dose increased, CRP concentration, a marker of inflammation, was reduced [22]. The study also reported that a quadratic relationship was observed with the consumption of cocoa flavanols and IL-6 when the dose of flavanols reached 400 mg.

### 3.5. Other Measurements

Other variables that were measured in some of the clinical trials in this review include cortisol [24]; flow-mediated dilation of the brachial artery, which is a marker of endothelial function, small and large artery elasticity [21], and quality of life [35]. Only two studies reported measuring compliance by using cocoa metabolite from urine samples [20,21].

## 4. Discussion

The main aim of this scoping review was to assess existing human studies on the effects of cocoa polyphenol or chocolate consumption on obesity-related outcomes (anthropometric measurements, glucose profiles, lipid profiles, and inflammatory and oxidative biomarkers) in obese subjects. Many clinical trials investigating the effects of cocoa polyphenols have shown favorable effects, but there are also conflicting results, so it is crucial to do further investigation to address the difference.

The studies included in this review incorporated different forms of cocoa (chocolate, beverage, and powder) that were administered either in a normal caloric diet or as part of a reduced-calorie diet or in combination with exercise. The outcomes for the majority of these studies in anthropometric measurements do not favor the weight-reducing role of polyphenols in obese individuals. In fact, two studies reported an increase in body weight after an intervention with cocoa plus 500 mL of skim milk [19,20]. In contrast, three studies found reductions in anthropometric measurements (either body weight, BMI, or waist circumference) [28,35,39]. The dosage of cocoa used in these studies was 100 to 645 mg of polyphenols and the trial duration was 4 to 18 weeks. Two of the studies incorporated cocoa consumption with an energy-restricted diet (ERD) as their active arm intervention. A systematic review and meta-analysis of the effects of cocoa/dark chocolate supplementation on body weight and BMI concluded that body weight was only reduced in trials of >8 weeks’ duration and with cocoa or chocolate supplementation of >30 g per day. People with BMI up to 25 kg/m^2^ showed more prominent changes in body mass index, waist circumference, and cholesterol level after consumption of flavanol-containing products, and this supports the claim that supplementation with these products may have a greater impact on these risk factors for overweight and obese people [40].

A possible explanation for the nonsignificant improvement in anthropometric measurements includes the different doses and forms of cocoa used in the interventions leading to different amounts of calories, sugar, and fat of the investigated products, as extra calories may have hampered the weight-reduction effects. In addition, the difference in polyphenol and fat content between cocoa and dark chocolate could have made their comparison inaccurate. Generally, cocoa powder contains higher amounts of total polyphenols, catechin, and epicatechin than dark chocolate but less fat [41]. On the other hand, dark chocolate that contains relatively high amounts of cocoa (50 to 85%) also contains high amounts of polyphenols (460–610 mg/kg) [42]; however, dark chocolate may also have high amounts of saturated fat and sugar [43]. Although many human studies have reported the health benefits of dark chocolate, the effects are limited by the calorie, sugar, and fat content; therefore, it must be introduced in the context of a well-balanced diet and controlled calories. In contrast, Almoosawi et al. [24] indicated that consumption of 20 g dark chocolate, providing 500 mg of polyphenols and a total of 102 kcal per day, by overweight and obese individuals is considered acceptable and can counteract the dietary impact on fat and energy content. The lipid content in cocoa is contributed by cocoa butter, a mixture of saturated and monounsaturated fatty acids. Although chocolate lipid content is relatively high, one-third of it is stearic acid, which is known to have non-atherogenic and natural effects on CVD. Stearic acid is unique in that it does not increase serum lipid levels to the same degree as other saturated fatty acids [44,45]. In addition, chocolate contains other nutrients that may be more beneficial to health than saturated fat content. Given the potential adverse effects on human health and the documented positive health benefits of chocolate consumption, it is important to enjoy chocolate in moderate quantities to avoid potential adverse effects.

Among all studies that reported body weight as their outcome measure, only one included body fat composition. The study showed significant reductions in both body weight and body fat. Further studies that include body fat measurements are needed to better understand the role of polyphenol intake in modulating body fat reduction, particularly by altering lipid metabolism, such as fatty acid oxidation [46] and synthesis [47]. On another note, the smell of chocolate has been reported to suppress appetite, which is inversely related to levels of ghrelin [48,49]. This finding suggests that cocoa polyphenol intake may be beneficial in reducing both appetite and weight gain; however, none of the intervention studies included in this scoping review reported any satiety- or peptide-related hormones (ghrelin, leptin, and adiponectin) as part of their outcome measures. This information may be helpful in further understanding the effects of cocoa consumption on body weight and body fat.

Cocoa polyphenols have been previously suggested to lower CVD risk by modulating the lipid profile and blood pressure. In this review, improvements in lipid profiles were mainly achieved in subjects with some risk of CVD, such as metabolic syndrome [19,27,34,35,50]. In contrast, Munguia et al. [35], in a study using cocoa powder extract for 4 weeks, reported a 23.4% increase in TG level. It was also noted that HDL-c level was significantly reduced by 17% when compared to placebo. We suspect that this was due to the time effect, as Leyva Soto et al. [34] reported reductions in TC, TG, and LDL-c as well as blood pressure after 6 months of daily intake of 2 g of dark chocolate. Interestingly, studies have also reported only increased HDL-c, but no improvement in TC, TG, and LDL-c [19,20,21]. Acute consumption of cocoa beverage providing 960 mg of polyphenols showed that postprandial HDL remained higher compared to placebo after 6 h in subjects with type 2 diabetes after consuming a high-fat breakfast [21].

The evidence on the relationship between the amount of cocoa or dark chocolate and serum lipids is still inconclusive. As for the experiments carried out on subjects without cardiovascular risk, the findings were controversial. Studies included in this review found no significant changes in serum lipids following consumption of 500 mg of polyphenols in dark chocolate for 2 weeks [25] or 4 weeks [24] in healthy obese individuals. A systematic review on the effect of cocoa on serum lipid reported consistent findings, whereas cocoa consumption was only effective in reducing LDL-c and TC among participants with some type of cardiovascular disease or metabolic risk factors, but not among healthy subjects [51]. In terms of flavanol dose, a systematic review and meta-analysis indicated that studies in which less than 500 mg of flavanol was administered daily showed greater LDL-c reduction compared to higher doses [52], concurring with Jia et al. [51], who showed an effective dose of less than 260 mg. This could be because a high amount of polyphenols can counteract their benefits to the lipid profile [51]. Similarly, Tokedo et al. [52] reported a saturation effect with a 500 mg daily dose; therefore a confirmatory study is needed to elucidate the optimal daily dose of flavanol for consumption.

Despite growing evidence from animal studies to support the anti-diabetic effects of some dietary polyphenols from cocoa extract [44,53,54,55], the present review of human studies indicated otherwise [38]. Dark chocolate and cocoa beverages containing 960 mg total polyphenols and 480 mg flavanols did not affect glucose and increased insulin levels in obese type 2 diabetes subjects given a high-fat breakfast [21]. A similar finding was reported in a study of subjects with hypertension and diabetes, in which consumption of 25 g of dark chocolate for 8 weeks did not improve insulin, fasting glucose, or HbA1c levels [54]. Current data are inadequate to suggest chocolate and cocoa for glycemic control due to contradictory findings in human intervention. Although few studies have suggested the protective effects of cocoa polyphenols on insulin resistance (IR) and blood glucose, the mechanisms involved remain unclear. This is partly due to a lack of understanding of the flavanols’ mechanisms of action [55].

Oxidative stress, indicated by excessive production of reactive oxygen species (ROS) in cells and tissues, plays an important role in the pathogenesis of insulin resistance [56], inflammation, and many chronic diseases [57]. Obesity is a dynamic multifactorial disorder characterized by excess adipose tissue mass arising through hypertrophy and hyperplasia of adipocytes [58], including the accumulation of ROS and the activation of cell inflammatory markers such as interleukin-6 (IL-6), tumor necrosis factor alpha (TNF-α), adiponectin, and leptin. Visceral adiposity is associated with greater production of these inflammatory adipocytokines, resulting in insulin resistance, systemic inflammation, and many obesity-related metabolic disorders [59].

Epidemiological studies indicate that daily intake of flavonoid-rich foods and beverages, such as cocoa, is correlated with decreased cardiovascular disease risk due to their natural antioxidant properties. The National Health and Nutrition Examination Surveys of US adults found that higher intake of flavonoid-containing food is associated with lower levels of CRP, an inflammatory marker [31]. Moreover, it was suggested that the intake of foods rich in polyphenols decreases low-level inflammation [60]. In this review, only eight intervention studies measured oxidative and/or inflammatory markers. No improvement in oxidative and inflammatory biomarkers was reported in studies involving obese subjects with a risk of CVD [19,20], elevated waist circumference and type 2 diabetes [21], or LDL-c [50], or in healthy obese subjects [38]. In contrast, Stote et al. [22], in a study comparing different doses of cocoa flavanols (30–900 mg) among 20 obese adults, showed significant reductions in biomarkers of inflammation and oxidative stress (CRP, total 8-isoprostane, and IL-6 concentration). The study also noted that the effects of cocoa consumption on IL-6 concentration was achieved with a flavanol dose of 400 mg.

The anti-inflammatory activity of cocoa depends largely on the dosage of flavanol. Findings in this review indicate variations in the dose of cocoa or chocolate flavanols because their content varies among products. In addition, the polyphenol content of cocoa also depends on its origin and the manufacturing process of the final product; therefore, the use of different cocoa products with different phenolic compositions might cause conflicting findings. Apart from flavonoids, other compounds such as dietary fiber and methylxanthines, particularly theobromine, must also be taken into account. Research that includes many aspects involving weight control such as peptide hormones, fatty acid metabolism, and related pathways is needed in order to understand and elucidate the effects of cocoa polyphenols in obese adults. Furthermore, there is increasing interest in studying the mechanisms of cocoa, such as its prebiotic effect in relation to its interaction with gut microbiota and health outcomes [61], but studies are scarce. None of the studies included in this review investigated the effects of cocoa on obese individuals by considering its effects on gut microbiota to further enhance the understanding of its putative anti-obesity activity.

In addition, a detailed and unbiased assessment of dietary exposure to cocoa products is required to further evaluate the relationship between cocoa consumption and health outcomes. The polyphenol content of the foods being examined and the structure of the polyphenol subclasses should also be included. Estimation of polyphenol intake, which is currently limited to data derived from food frequency questionnaires, is also problematic as it is subjected to recall bias and over- or underestimation. Knowing which metabolites are being produced upon cocoa ingestion is also important, as this knowledge will help us understand its absorption, distribution, and metabolism.

## 5. Conclusions

In conclusion, selected studies in this review suggest that more studies are needed to investigate the impact of cocoa polyphenol intake on obese adults, as the results of the included studies are still inconclusive. More studies are needed among healthy obese individuals, as very limited studies have been done on this population; most studies have involved obese subjects with some risk of CVD. This would promote further exploration of the exact mechanism by which cocoa polyphenols, mainly flavanols, exert their beneficial effects on obesity and in preventing the risk of CVD. Long-term research involving a broad cohort and diet and exercise controls is required to validate the possible impact of polyphenols on obesity before recommending cocoa or dark chocolate rich in polyphenols in the diet for managing weight and obesity.

**Table 2:** Summary of studies assessing the effects of cocoa/dark chocolate on obesity in adults.

Study (Country)	Study Design	Participants	Dose/Form of Cocoa	Duration	Outcome Measures
Davison et al. [26] (Australia)	Randomized double-blind parallel placebo-controlled trial	*N* = 49; (age 18–65) (17 males, 32 females)	G1: High flavanol (902 mg) + 45 min exercise 3 times a week G2: Low flavanol (36 mg) + 45 min exercise 3 times a week G3: High flavanol (902 mg) G4: Low flavanol (36 mg) Form of cocoa: dairy-based powder mix	12 weeks	High flavanol increased FMD acutely and chronically ↓ Insulin resistance by 0.31%, diastolic BP by 1.6 mmHg in high flavanol group independent of exercise No significant effects on BMI, waist circumference, total body fat for all groups
Monagas et al. [20] (Spain)	Randomized cross-over controlled trial	*N* = 42 at high risk for CVD (age > 55) (19 men, 23 women)	G1: 40 g cocoa powder (495 mg polyphenols) + 500 mL skim milk/day G2: 500 mL skim milk/day	4 weeks	↑ BW in G1 No changes in SBP, DBP, heart rate in both groups; no changes in FBG, TC, TG, LDL ↑ HDL in G1 ↓ Inflammatory markers ICAM-1 in G1 No changes in IL-6, HsCRP in both groups
Almoosawi et al. [25] (UK)	Randomized cross-over controlled trial	*N* = 14 (age 21–50) (8 males, 6 females)	G1:20 g dark chocolate (500 mg polyphenols) G2: 20 g dark chocolate (1000 mg polyphenols)	2 weeks 1 week run-in phase, 1 week washout	No changes in BMI, WC in both groups ↓ FBG, SBP, DBP in both groups; no significant difference between groups No changes in BW, BMI, WC in both groups No changes in TC in both groups
Njike et al. [38] (USA)	Randomized controlled double-blind cross-over trial	*N* = 44	G1: sugar-free cocoa beverage (22 g cocoa, 805 mg flavanol) G2: sweetened cocoa beverage (22 g cocoa, 805 mg flavanol) G3: placebo beverage	6 weeks 4 weeks washout	FMD ↑ in G1, G2 compared to placebo No changes in BW, BMI, BP No changes in TG, LDL-c, HDL-c No changes in FBG, CRP, LDL oxidation
Khan et al. [19] (Spain)	Randomized controlled cross-over trial	*N* = 42 at high risk of CVD (age > 55) (19 men, 23 women)	G1: 40 g cocoa powder (495 polyphenols) + 500 mL skim milk G2: 500 mL skim milk	4 weeks	↑ HDL-c, ↓ oxLDL in G1 No changes of TC, TG, LDL-c ↑ Body weight in both groups No changes in BP, heart rate
Stote et al. [22] (USA)	Randomized placebo-controlled cross-over trial	*N* = 20 obese at risk of insulin resistance (age 25–55) (10 women, 10 men)	G1: control, 56 g cocoa powder beverage (30 mg flavanol) G2: low, 56 g cocoa powder beverage (180 mg flavanol) G3: medium, 56 g cocoa powder beverage (400 mg flavanol) G4: high, 56 g cocoa powder beverage (900 mg flavanol) G5: tea (900 mg)	5 days intervention 10 days washout	No changes in glucose, insulin, total AUC for glucose, HOMA-IR, ISI, QUICKI Total 8-isoprostane, CRP, IL-6 ↓in all cocoa treatments; with increased dose, all parameters decreased
Almoosawi et al. [24] (UK)	Randomized controlled single-blind cross-over trial	*N* = 42 (21 normal BMI, 21 owt/obese)	G1: 20 g dark choc (500 mg polyphenols) G2: 20 g placebo dark choc	4 weeks 2 weeks washout period	↓ SBP, DBP No changes in fasting insulin No changes in lipid profile No changes in body weight, BMI No changes in WC, WHR
West et al. [23] (USA)	Randomized placebo-controlled cross-over trial	*N* = 30 postmenopausal women (age 40–64)	G1: 37 g dark choc + sugar-free cocoa beverage, total flavanol 814 mg G2: low-flavanol chocolate bar + cocoa-free beverage, total flavanol 3 mg	4 weeks 2 weeks washout period	↑ Basal diameter and peak diameter of brachial artery, basal blood flow No changes in body weight, BMI, waist circumference, hip circumference, or WHR No changes in blood glucose, blood lipid profile
Nickols Richardson et al. [28] (USA)	Randomized controlled parallel trial	*N* = 60 overweight/obese women (age 25–45)	Daily energy-restricted diet with: G1: 236 mL sugar-free natural cocoa beverage per day (272 kJ/day), one 1.45 oz dark chocolate (270 mg flavanol) G2: 236 mL sugar-free cocoa-free vanilla beverage per day (272 kJ/day), two non-chocolate sweet snacks (fruit-flavored licorice stick) (0 mg flavanol)	18 weeks	↓ BW, SBP, DBP, glucose, insulin No change in lipid profile
Basu et al. [21] (USA)	Randomized controlled double-blind cross-over trial	*N* = 18 obese adults with elevated waist circumference, type 2 diabetes (14 females, 4 males, age > 21)	G1: 20 g cocoa beverage (960 mg polyphenols, 480 mg flavanols) + high-fat fast-food breakfast (766 kcal) G2: 12 g flavanol-free placebo (110 mg polyphenols, <0.1 mg flavanols) + high-fat fast-food breakfast (766 kcal)	6 h 1 week washout	↑ HDL-c, serum insulin No changes in TC, LDL-c, glucose, HsCRP No changes in SBP, DBP, HOMA-IR, small artery elasticity Lower large artery elasticity
Munguia et al. [35] (Mexico)	Randomized controlled double-blind trial	*N* = 15 overweight with borderline criteria of metabolic syndrome (age 20–60) (11 females, 4 males)	G1: cocoa bean extract powder (80 mg flavonoids) G2: placebo powder (sugar-free, no flavonoids)	4 weeks	↓ BW, BMI, WC ↓ SBP ↓ FBG ↓ TC, LDL-c, HDL-c, TG ↓ Oxidative marker: MDA
Ibeiro-Baraibar et al. [27] (Spain)	Randomized controlled parallel double-blind trial	*N* = 24 (12 males, 12 females) (age 50–80)	G1: –15% energy-restricted diet + ready-to-eat meals + 1.4 g cocoa extract (645 mg polyphenols) G2: –15% energy-restricted diet + ready-to-eat meals	4 weeks	↓ Body weight in both groups No changes in insulin level, fasting glucose for both groups ↓ TC, TG, LDL-c, HDL-c) in both groups ↓ oxLDL, MPO, ICAM in G1
Lee et al. [50] (USA)	Randomized controlled four-period cross-over trial	*N* = 31 overweight/obese with elevated LDL-c (13 males, 18 females) (age 30–70)	G1: No treatment food (average American diet) G2: 42.5 g almonds (ALD)G3: 18 g cocoa powder + 43 g dark chocolate (CHOC) (422 mg polyphenols) G4: ALD + CHOC (422 mg polyphenols)	4 weeks 2 weeks washout period	CHOC + ALD ↓ apolipoprotein B ↓TC and LDL-c in G2 Higher FBG in G3 and G4, no changes in insulin, IR, hsCRP Oxidative stress: No changes in all groups (LDL oxidation, urinary 8-isoprostane)
Leyva Soto et al. [34] (USA)	Randomized placebo-controlled double-blind trial	*N* = 84 young volunteers (47 men, 37 women)	G1: 2 g dark chocolate (70% cocoa) G2: 2 g milk chocolate	6 months	↓ TC, TG, LDL-c ↓ WC ↓ FBG, HOMA-IR ↓ BP

FMD, flow-mediated dilation; BMI, body mass index; BW, body weight; WC, waist circumference; WHR, waist-to-hip ratio; BP, blood pressure; DBP, diastolic blood pressure; SBP, systolic blood pressure; FBG, fasting blood glucose; TC, total cholesterol; TG, triglycerides; LDL-c, low-density lipoprotein cholesterol; HDL-c, high-density lipoprotein cholesterol; CRP, C-reactive protein; HsCRP, high sensitive C-reactive protein; HOMA-IR, Homeostatic Model Assessment for Insulin Resistance; QUICKI, quantitative insulin sensitivity check index; IL-6, interleukin 6; ICAM-1, intercellular adhesion molecule 1; MDA, malondialdehyde. ↓, decreased; ↑, increased.

**Table 3:** Characteristics and outcomes of observational studies.

Study	Population	Investigation	Outcomes
Vernarelli and Lambert [31]	9551 adults	Association of flavonoid consumption and multiple markers for obesity including body mass index, waist circumference, and C-reactive protein	An inverse association between total flavonoid intake and BMI (body mass index) was observed (p-trend, 0.013) after adjusting for age, sex, race, education, physical activity, smoking status, poverty/income ratio, total alcohol intake, total fat intake, and dietary energy density
Cuenca-Garcia M et al. [29]	1458 adolescents	To determine whether chocolate consumption is associated with higher BMI and other markers of total and central body fat	Higher consumption of chocolate was associated with lower BMI, body fat estimated from skinfold and BIA (bioelectrical impedance analysis), waist circumference
O’Neil et al. [62]	15,023 adults	To determine candy and chocolate consumption with nutrient intake, diet quality, weight status, and CVD (cardiovascular disease) risk factors	Chocolate consumers had lower weight, TG (triglycerides) and CRP (C-reactive protein) levels, and higher HDL-c (high-density lipoprotein) levels
Golomb et al. [32]	1018 adults	To examine the cross-sectional relationship of chocolate consumption frequency and BMI	Frequent chocolate intake linked to lower BMI
Greenberg and Buijsse [33]	15,732 and 12,830 participants at the first and second visit	To assess prospective and cross-sectional associations between chocolate intake and body weight	Prospective analysis shows more frequent consumption of chocolate was significantly associated with long-term greater weight gain in a dose-response manner; cross-sectional analysis yielded the opposite: an inverse association between chocolate intake and current BMI

## Figures and Tables

**Table 1:** Inclusion and exclusion criteria.

Criteria	Inclusion	Exclusion
Literature type	Journal (research articles)	Systematic review, book and book series, book chapter, conference proceedings
Language	English	Non-English
Timeline Study type	Between 2008 and 2019 Human clinical trials and observational studies	Published before 2008 Animal and in vitro studies
